# The importance of mineralogical composition for the cytotoxic and pro-inflammatory effects of mineral dust

**DOI:** 10.1186/s12989-022-00486-7

**Published:** 2022-07-06

**Authors:** Vegard Sæter Grytting, Magne Refsnes, Marit Låg, Eyolf Erichsen, Torkil Sørlie Røhr, Brynhild Snilsberg, Richard Aubrey White, Johan Øvrevik

**Affiliations:** 1 Department of Air Quality and Noise, Division of Climate and Environmental Health, Norwegian Institute of Public Health, Skøyen, PO Box 222, 0213 Oslo, Norway; 2 Geological Survey of Norway, Trondheim, Norway; 3 Norwegian Public Roads Administration, Trondheim, Norway; 4 Department of Method Development and Analytics, Division of Infection Control and Environmental Health, Norwegian Institute of Public Health, Oslo, Norway; 5 Division of Climate and Environmental Health, Norwegian Institute of Public Health, Oslo, Norway; 6 Department of Biosciences, Faculty of Mathematics and Natural Sciences, University of Oslo, Oslo, Norway

**Keywords:** Particulate matter, Mineral particles, Stone particles, Quartz, Silica, Macrophages, Epithelial cells, Inflammation

## Abstract

**Background:**

Respirable mineral particles represent a potential health hazard in occupational settings and ambient air. Previous studies show that mineral particles may induce cytotoxicity and inflammatory reactions in vitro and in vivo and that the potency varies between samples of different composition. However, the reason for these differences is largely unknown and the impact of mineralogical composition on the biological effects of mineral dust remains to be determined.

**Methods:**

We have assessed the cytotoxic and pro-inflammatory effects of ten mineral particle samples of different composition in human bronchial epithelial cells (HBEC3-KT) and THP-1-derived macrophages, as well as their membranolytic properties in erythrocytes. Moreover, the results were compiled with the results of recently published experiments on the effects of stone particle exposure and analysed using linear regression models to elucidate which mineral components contribute most to the toxicity of mineral dust.

**Results:**

While all mineral particle samples were more cytotoxic to HBEC3-KT cells than THP-1 macrophages, biotite and quartz were among the most cytotoxic in both cell models. In HBEC3-KT cells, biotite and quartz also appeared to be the most potent inducers of pro-inflammatory cytokines, while the quartz, Ca-feldspar, Na-feldspar and biotite samples were the most potent in THP-1 macrophages. All particle samples except quartz induced low levels of membranolysis. The regression analyses revealed associations between particle bioactivity and the content of quartz, muscovite, plagioclase, biotite, anorthite, albite, microcline, calcite, chlorite, orthopyroxene, actinolite and epidote, depending on the cell model and endpoint. However, muscovite was the only mineral consistently associated with increased cytotoxicity and cytokine release in both cell models.

**Conclusions:**

The present study provides further evidence that mineral particles may induce cytotoxicity and inflammation in cells of the human airways and that particle samples of different mineralogical composition differ in potency. The results show that quartz, while being among the most potent samples, does not fully predict the toxicity of mineral dust, highlighting the importance of other particle constituents. Moreover, the results indicate that the phyllosilicates muscovite and biotite may be more potent than other minerals assessed in the study, suggesting that this group of sheet-like minerals may warrant further attention.

**Supplementary Information:**

The online version contains supplementary material available at 10.1186/s12989-022-00486-7.

## Introduction

Minerals are groups of inorganic solid chemical compounds that may exist in a pure form or as aggregates in rocks [1]. The largest group of minerals is the silicates, which make up the majority of the earth’s crust in the form of feldspars, quartz, pyroxenes, amphiboles, micas and clay minerals [1]. Many minerals are commercially exploited and are used in a wide range of applications, including construction, ceramics, paints, fillers, plastics, electronics and abrasives [2]. Consequently, inhalation of mineral particles represents a potential health hazard in industries and occupations where rocks and minerals are mined, processed and handled [3–6]. In addition, crustal material is a common constituent of respirable ambient particulate matter, a source of exposure for the general population [7–10]. In urban areas, traffic generates coarse mineral-rich particles from abrasion of road surfaces and causes resuspension of accumulated road dust [11, 12]. This is especially problematic in Nordic countries, where the contribution of particles from road surface wear can be high due to the prevalent use of studded tyres during winter and spring [11, 13–15]. However, while exposure to quartz and asbestos particles is a well-characterized health hazard associated with progressive fibrotic lung disease and cancer [16–18], the potential toxicity of other minerals has received considerably less attention both in occupational and environmental settings.

Induction of pulmonary inflammation is regarded as a key event in the adverse health effects of inhaled particles and may involve particle-induced oxidative stress, interaction with cellular membranes, activation of cell-surface receptors, and direct interactions with intracellular molecular targets [19]. Previous studies from our group show that stone particle samples of different mineralogical and metal composition differ in their ability to induce inflammatory cytokines both in vitro and in vivo [20–27]. However, the reasons for these differences remain to be clarified, and the pro-inflammatory potency of the samples could not be attributed to differences in particle surface area, particle-induced reactive oxygen species (ROS) or soluble metal constituents [21, 22, 26]. Although early studies did not identify which particle characteristics were linked to the pro-inflammatory properties, the results seemed to indicate that particle samples with high content of feldspar minerals were the least potent [20, 23–25, 27]. Conversely, our recent study suggests that some feldspar-rich stone particle samples may induce cytotoxicity and acute pro-inflammatory responses to a similar or greater extent than quartz [21].

In the present study, the role of mineralogical composition on the inflammatory potential of mineral dust was examined further. The pro-inflammatory and cytotoxic effects of exposure to ten mineral particle samples were assessed in human bronchial epithelial cells and macrophage-like cells. Furthermore, the ability of the particles to induce lysis of human red blood cells was assessed to explore membranolytic properties. Finally, the data from the present study was compiled with the results from Grytting et al. [21], where the responses to several stone particle samples with different mineralogical composition were assessed, and analysed using linear regression models to assess the potential association between mineralogical composition and the different cellular endpoints. The results provide further evidence that mineral particles may induce cytotoxicity and inflammation in cells of the human airways. Moreover, the potential involvement of certain mineral components is highlighted.

## Results

### Particle sample characteristics

**Fig. 1:**
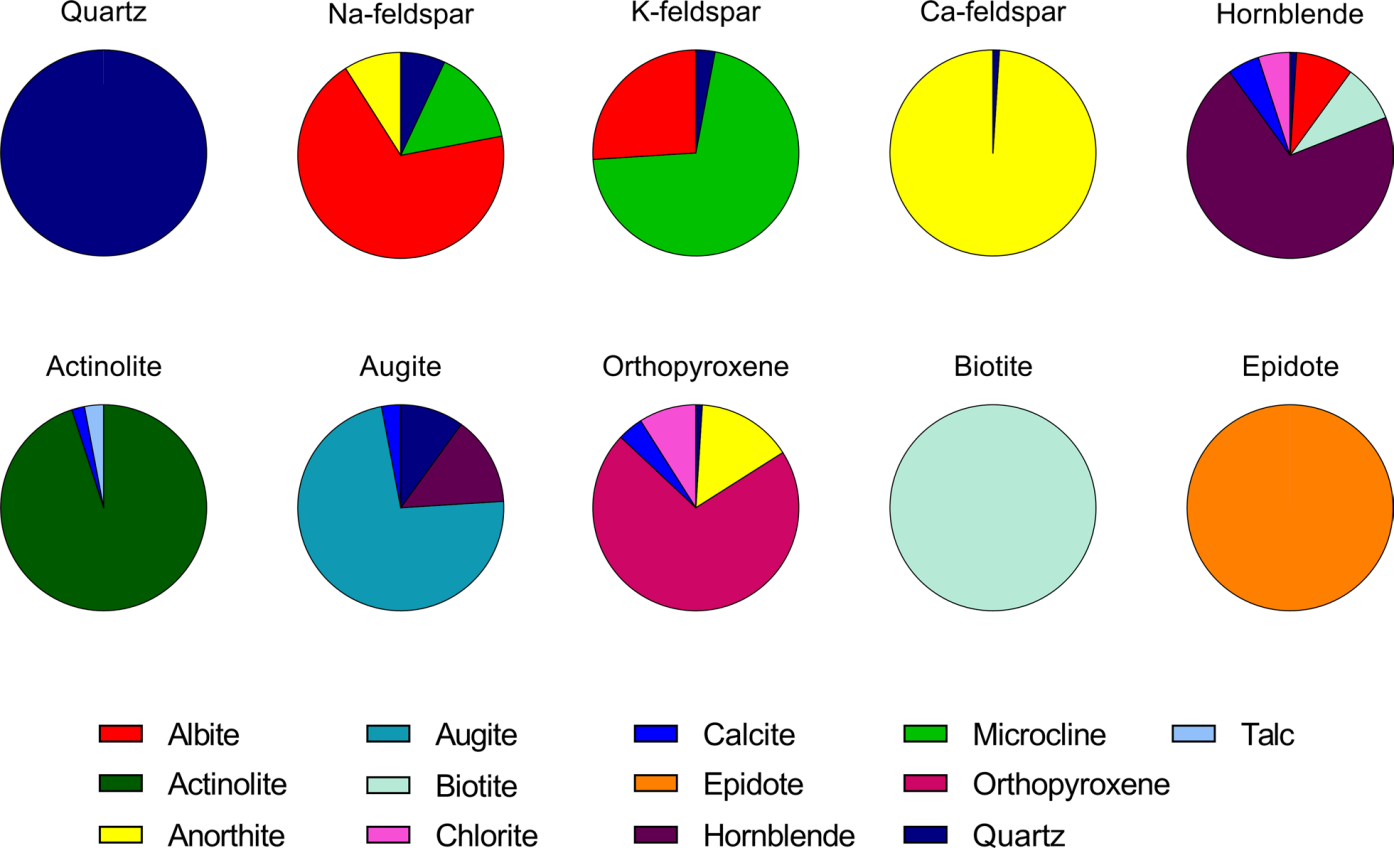
Mineralogical composition. The mineralogical composition of the Na-feldspar, K-feldspar, Ca-feldspar, biotite, epidote, augite, actinolite, hornblende, quartz and orthopyroxene samples was determined using X-ray diffraction analysis. The results are presented as percentages of total and are also presented in Additional file 1: Table S1

Ten different mineral samples were chosen for the present study to include the most common rock-forming classes of silicates and to represent the major mineral components of the particle samples studied in Grytting et al. [21] (Fig. 1, Additional file 1: Table S1). The mineral samples are classified according to their major mineral constituents (Table 1). Four samples of tectosilicates were included; one sample of quartz as well as three samples of feldspar minerals, termed N-feldspar, Ca-feldspar and K-feldspar, which were chosen to represent the Na-, Ca- and K-endmembers, albite, anorthite and microcline, respectively (Additional file 2: Fig. S1). Four inosilicate samples were also included in the study; hornblende and actinolite, which are part of the amphibole group of inosilicates, and augite and orthopyroxene, which belong to the pyroxene group. Biotite is a mica mineral and was included as a representative phyllosilicate, a group of minerals characterized by their sheet-like morphology. A sample of the sorosilicate epidote was also included in the study.

**Table 1:** Mineralogical classification mineral particle samples

Sample	Mineralogical classification
Quartz	Tectosilicates, quartz group
Na-feldspar	Tectosilicates, feldspar group
K-feldspar	Tectosilicates, feldspar group
Ca-feldspar	Tectosilicates, feldspar group
Hornblende	Inosilicates, amphibole group
Actinolite	Inosilicates, amphibole group
Augite	Inosilicates, pyroxene group
Orthopyroxene	Inosilicates, pyroxene group
Biotite	Phyllosilicates, mica group
Epidote	Sorosilicates, epidote group

#### Mineralogical composition

X-Ray diffraction (XRD) analysis of the mineral samples revealed a varying degree of purity in terms of mineralogical composition (Fig. 1, Additional file 1: Table S1). Quartz, biotite and epidote were pure mineral samples, while the actinolite sample also contained small amounts of calcite and talc. Of the feldspar samples, the Ca-feldspar sample was the purest, consisting of 99% anorthite, whereas the Na- and K-feldspar samples consisted of 69% albite and 71% microcline, respectively, in addition to small amounts of quartz and up to 26% of other feldspar minerals. The hornblende sample contained smaller amounts of quartz, albite, biotite, calcite and chlorite, while the augite sample also contained 10% quartz, 14% hornblende and small amounts of calcite. The orthopyroxene sample contained 15% anorthite, as well as small amounts of quartz, calcite and chlorite.

The mineralogical composition of the quartzite, anorthosite, rhomb porphyry, dacite, quartz diorite, hornfels and α-quartz samples included in the statistical analyses below has been described previously [21], but is also included in the online supplement of the present study (Additional file 1: Table S1). All stone particle samples were generated from crushed rock materials consisting of an assemblage of different minerals, while the α-quartz sample (Min-U-Sil 5) is a commercially available quartz sample of high purity [21]. Quartz and feldspar minerals were the most common mineral constituents, while smaller amounts of biotite, epidote, hornblende, augite, calcite, chlorite and muscovite were detected in one or more of the samples. The feldspar was either in the form of microcline or plagioclase. The term plagioclase is used to describe solid solutions of albite and anorthite. It should be noted that there is some uncertainty whether the K-feldspar in the rhomb porphyry sample is in the form of microcline or orthoclase, as the high similarity of the minerals makes it hard to differentiate between them. For the purpose of the statistical analyses in the present study, the K-feldspar content is assumed to consist of microcline as for the other particle samples (Additional file 1: Table S1).

#### Elemental composition

The elemental composition of the mineral particle samples was determined by X-ray fluorescence (XRF) analysis and is presented in Table 2. The elemental compositions were in agreement with the mineralogy of the samples (Additional file 1: Table S2), with the quartz sample consisting almost entirely of silicon, while aluminium, calcium, sodium and potassium were major constituents in the feldspar samples. The biotite, epidote, actinolite, augite, hornblende and orthopyroxene samples also contained significant quantities of iron and magnesium. Smaller amounts of vanadium, chromium, barium, cobalt, nickel, lead, zinc and zirconium were detected in one or more of the particle samples. Consistent with the tested minerals being silicates, silicon was a primary constituent in all samples.

**Table 2:** Elemental composition (%) of the quartz, Na-feldspar, K-feldspar, Ca-feldspar, hornblende, actinolite, augite, orthopyroxene, biotite and epidote samples

	Quartz	Na-feldspar	K-feldspar	Ca-feldspar	Hornblende	Actinolite	Augite	Orthopyroxene	Biotite	Epidote
SiO_2_	98.6	66.5	65.2	48.5	45.0	55.8	56.7	49.1	34.2	37.5
Al_2_O_3_	0.025	19.5	18.7	32.2	9.1	0.557	0.935	8.05	19.1	22.6
Fe_2_O_3_	0.019	0.176	0.121	0.259	14.5	8.23	6.96	17.1	25.2	13.8
TiO_2_	–	0.011	0.012	0.026	0.099	0.031	0.232	0.731	2.53	0.092
MgO	0.11	–	–	0.067	12.2	20.0	13.6	20.4	4.54	0.092
CaO	–	1.69	0.358	14.5	10.8	12.6	18.2	3.02	0.671	22.6
Na_2_O	–	7.04	2.57	2.51	1.75	0.15	0.94	0.34	0.3	–
K_2_O	0.013	3.96	12.3	0.258	1.34	0.039	0.072	0.126	8.24	0.025
MnO	–	–	–	–	0.071	0.108	0.067	0.235	0.348	0.195
P_2_O_5_	–	0.017	0.023	0.013	0.014	0.017	0.012	0.016	0.603	0.011
BaO	–	–	0.026	0.012	–	–	–	–	–	–
Co_2_O_3_	–	–	–	–	–	–	–	0.012	–	–
Cr_2_O_3_		–	–	–				0.062	0.012	0.019
CuO	–	–	–	–	–	–	–	–	–	–
NiO	–	–	–	–	–	–	–	0.024	–	–
PbO	–	–	0.009	–	–	–	–	–	–	–
SrO	–	0.007	0.007	0.025	0.006	–	–	0.007		0.086
V_2_O_3_		–	–	–	–	–	0.026	0.035	0.017	0.077
ZnO	–	–	–	–	–	–	–	0.017	0.161	–
ZrO_2_	–	–	–	–	0.013	–	0.015	–	0.128	–

#### Endotoxin contamination

Adhered endotoxin has been shown to be an important contributor to the inflammatory effects of ambient particles [28, 29] and could possibly confound the results of the mineral particle exposure. Thus, the content of bacterial endotoxin was determined by the limulus amebocyte lysate (LAL) assay. The endotoxin concentration did not exceed 1 EU/mg for any of the mineral particle samples (Table 3). Importantly, no statistically significant associations were found between endotoxin concentration and the endpoints presented in the subsequent sections, suggesting that the endotoxin content of the samples had negligible impact (Data not shown).

**Table 3:** Endotoxin contamination

Particle sample	Endotoxin content (EU/mg)
Quartz	0.636
Na-feldspar	0.979
K-feldspar	0.921
Ca-feldspar	0.948
Hornblende	0.914
Actinolite	0.411
Augite	0.765
Orthopyroxene	0.744
Biotite	0.588
Epidote	0.674

#### Particle size distribution

**Fig. 2:**
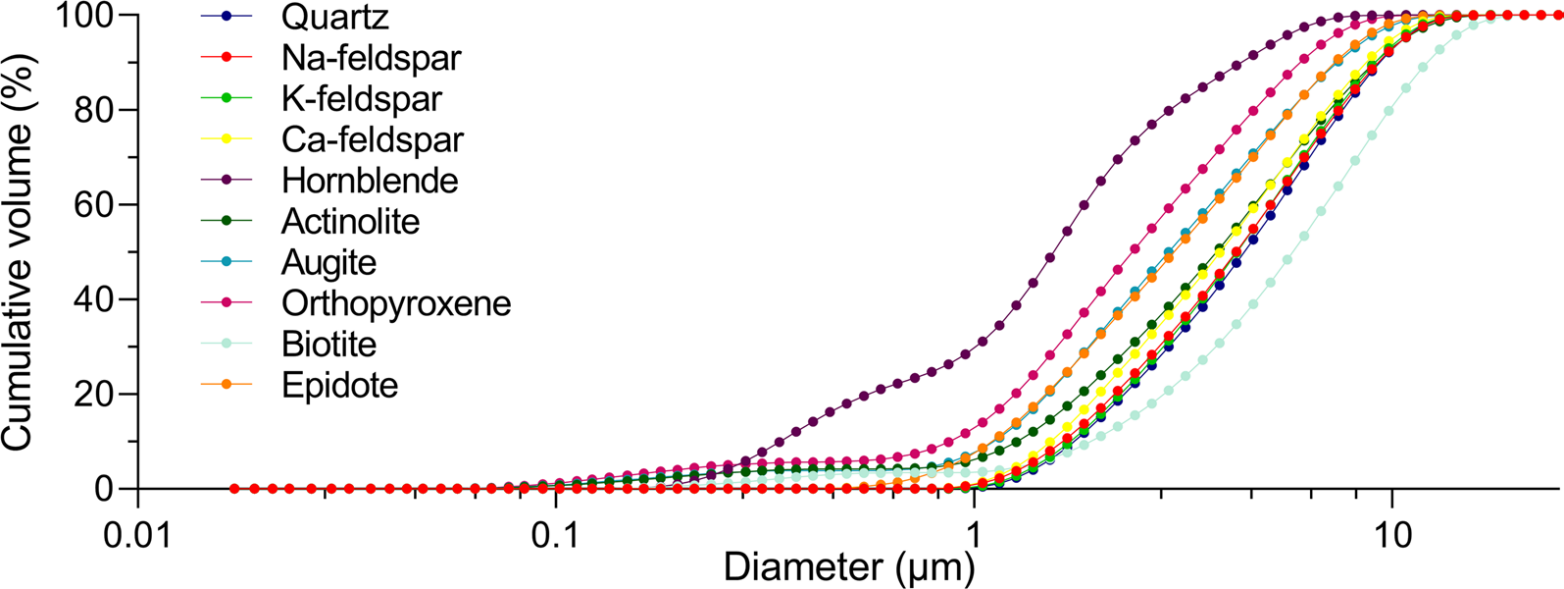
Particle size distribution. The size distributions of the Na-feldspar, K-feldspar, Ca-feldspar, biotite, epidote, augite, actinolite, hornblende, quartz and orthopyroxene samples were determined using coulter counter analysis. The results represent cumulative volume and are presented as percentages

The size distributions of the particle samples were determined by coulter counter analysis and are presented in Fig. 2. For all particle samples except biotite, over 95% of the particles were below 10 µm in diameter. The highest amount of large particles was detected in the biotite sample with approximately 55% of the particles being over 5 µm and 15% over 10 µm. Conversely, the hornblende sample had the highest amount of small particles with over 75% of the sample being below 2.5 µm and 31% below 1 µm. The orthopyroxene sample also contained a large amount of small particles with approximately 55% of the particles being below 2.5 µm and 14% below 1 µm. The quartz, Na-feldspar, K-feldspar, Ca-feldspar, epidote, actinolite and augite samples exhibited intermediate particle size distributions, with somewhat smaller particles in the epidote and augite samples.

### Cell viability

**Fig. 3:**
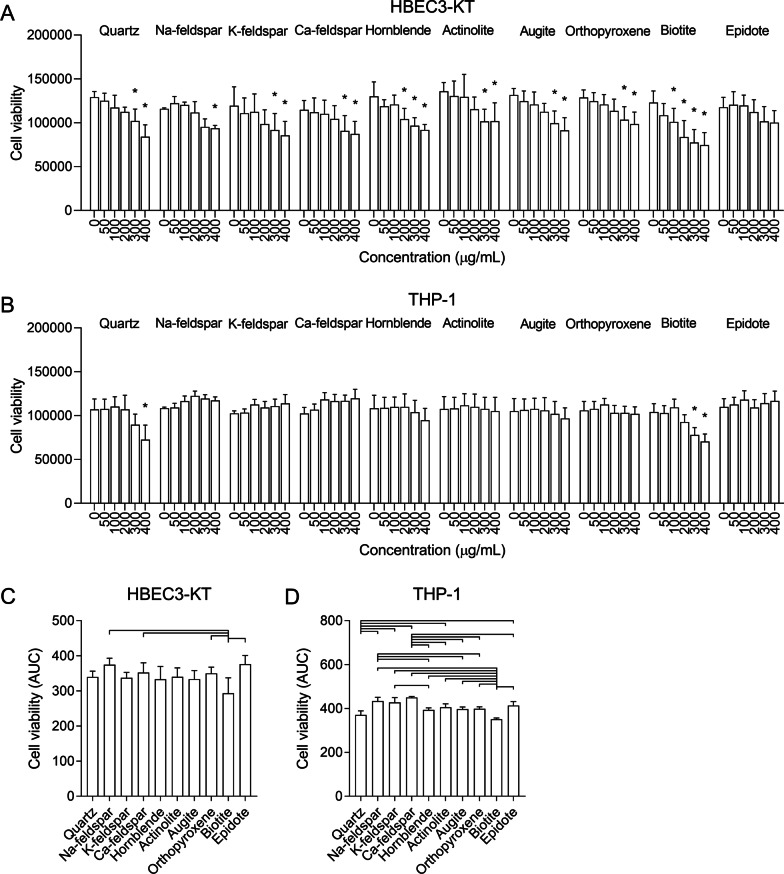
Mineral particles induce sample- and concentration-dependent decreases in cell viability. HBEC3-KT (**A** and **C**), THP-1 macrophages (**B** and **D**) were exposed to 0–400 µg/mL Na-feldspar, K-feldspar, Ca-feldspar, biotite, epidote, hornblende, actinolite, augite, quartz and orthopyroxene for 24 h. Cell viability was determined by alamarBlue © assay. Results are presented as mean ± SD (*n* = 4–5). Area under the curve (AUC) values in **C** and **D** were calculated from the values in A and B for the concentration-range of 0–400 µg/mL. All values were normalized to account for differences in baseline before calculating the AUC values by dividing each value by its respective control. Statistical significance is based on a two-way ANOVA followed by Dunnet’s post-test (**A** and **B**) or a one-way ANOVA followed by Tukey post-test (**C** and **D**). Asterisks (*) indicate statistically significant difference from the respective control (**A** and **B**), while a capped line indicates statistically significant difference between samples (**C** and **D**)

The particle-induced cytotoxicity varied considerably between the different samples and cell models (Fig. 3). In HBEC3-KT cells, all particle samples except epidote reduced cell viability to levels that were significantly different from control (Fig. 3A). The biotite sample was the most cytotoxic, causing a significant reduction in cell viability at 100 µg/mL, and a total reduction of approximately 40% at 400 µg/mL (Fig. 3A). The rest of the particle samples caused similar reductions in cell viability but reached statistical significance at different concentrations. When comparing particle samples based on area under the curve (AUC) values, the viability of cells exposed to the biotite sample was significantly lower than cells exposed to Na-feldspar, Ca-feldspar, epidote and orthopyroxene (Fig. 3C).

Compared with HBEC3-KT cells, the effect of particle exposure on cell viability was considerably lower in THP-1 macrophages (Fig. 3B). Only the biotite and quartz samples caused significant reductions in cell viability in THP-1 macrophages, reaching statistical significance at 300–400 µg/mL. At the highest concentration of 400 µg/mL, both the biotite and quartz samples reduced cell viability with approximately 30% (Fig. 3B). When comparing AUC values, biotite caused a significantly greater reduction in cell viability than all other particle samples except quartz. Quartz was significantly more cytotoxic than the Na-feldspar, K-feldspar, Ca-feldspar, epidote and actinolite samples (Fig. 3D). The feldspar samples seem to increase the viability, possibly due to increased metabolic activity of the cells (Fig. 3B), which likely accounts for the significant difference between these particle samples and other samples of low potency (Fig. 3D).

### Release of pro-inflammatory cytokines

Our previous work indicated that different stone particle samples may induce secretion of pro-inflammatory cytokines in a concentration-dependent manner [21]. Thus, we next assessed whether different mineral samples, representing the individual mineral components of the previously tested stone particle samples [21], could induce pro-inflammatory responses in a similar manner.

**Fig. 4:**
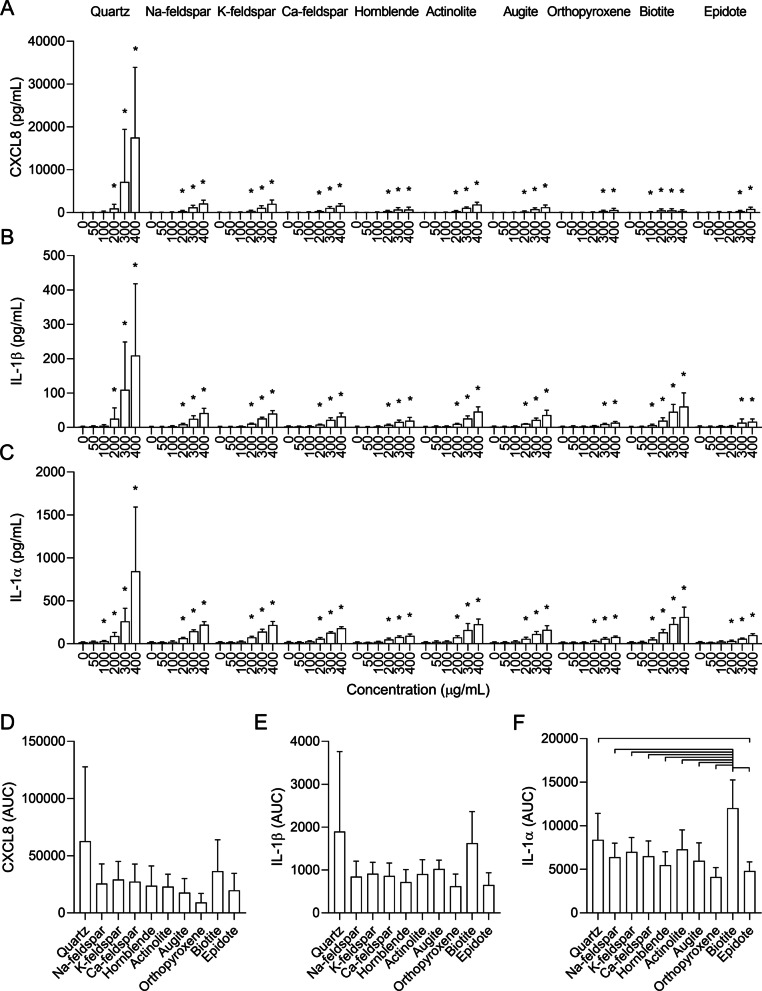
Mineral particles induce release of pro-inflammatory cytokines in human bronchial epithelial cells. HBEC3-KT cells were exposed to 0–400 µg/mL Na-feldspar, K-feldspar, Ca-feldspar, biotite, epidote, hornblende, actinolite, augite, quartz and orthopyroxene for 24 h. The release of CXCL8 (**A** and **D**), IL-1β (**B** and **E**) and IL-1α (**C** and **F**) in the cell culture supernatant was measured by ELISA. Results are presented as mean ± SD (*n* = 4–6). Area under the curve (AUC) values in **D**–**F** were calculated from the values in **A**–**C** for the concentration-range of 0–200 µg/mL. Statistical significance is based on a two-way ANOVA followed by Dunnet’s post-test (**A**–**C**) or a one-way ANOVA followed by Tukey post-test (**D**–**F**). Values in **A**–**E** were log-transformed prior to statistical analysis to satisfy model assumptions. Asterisks (*) indicate statistically significant difference from the respective control (**A**–**C**), while a capped line indicates statistically significant difference between samples (**D**–**F**)

All particle samples induced concentration-dependent increases in CXCL8, IL-1β and IL-1α in HBEC3-KT cells (Fig. 4). The biotite sample caused a significant increase at the lowest concentration, starting at 100 µg/mL for all cytokines. Na-feldspar, K-feldspar, Ca-feldspar, hornblende, actinolite and augite induced similar responses, reaching statistical significance at 200 µg/mL for all cytokines. Epidote and orthopyroxene were the least potent particle samples and induced significant increases in CXCL8 and IL-1β secretion at 300 µg/mL, and significant increases in IL-1α at 200 µg/mL. Compared with the other minerals, quartz induced the highest maximum levels for all cytokines, which were statistically significant at 200 µg/mL for CXCL8 and IL-1β, and at 100 µg/mL for IL-1α.

Due to the differences in cytotoxicity at the highest concentrations (Fig. 3A and C), the concentration-range of 0–200 µg/mL was chosen for between-sample comparison of AUC values to avoid underestimating the pro-inflammatory responses of the most cytotoxic particle samples. Nevertheless, it should be noted that biotite still induced an appreciable reduction in cell viability at 200 µg/mL (Fig. 3A). When comparing the AUC values in this concentration-range, most of the apparent differences between the samples were non-significant, although a similar order of potency was detected for all cytokines (Fig. 4D–F). While the responses induced by the biotite and quartz samples were slightly elevated, no statistically significant differences could be detected between the particle-induced responses for CXCL8 and IL-1β (Fig. 4D and E). For IL-1α, biotite induced a significantly higher response than all the other particle samples except quartz, while quartz induced a significantly higher response than orthopyroxene (Fig. 4F). It should be noted that the differences between quartz and the other particle samples would have been greater if the whole range of concentrations had been considered. Quartz induced considerably higher maximum levels of all cytokines than the other mineral samples (Fig. 4A–C). However, the effect was mostly associated with the highest concentrations of 300 and 400 µg/mL, which were not included when calculating the AUC values.

As shown in Fig. 2, there were marked differences in particle size distribution between some of the mineral particle samples. To assess whether this may have influenced the results, the correlation between the particle size and particle-induced cytokine release, expressed as mean AUC values (Fig. 4), was assessed using Pearson correlation. Significant positive correlations were detected between particle size and release of IL-1β (*r* = 0.6483, *p* = 0.0426) and IL-1α (*r* = 0.8393, *p* = 0.0024) in HBEC3-KT, showing that the mineral particle samples containing the largest particles were the most potent inducers of these cytokines. However, no significant association was detected between particle size and release of CXCL8 (*r* = 0.5317, *p* = 0.1137) (Additional file 3: Fig. S2a).

**Fig. 5:**
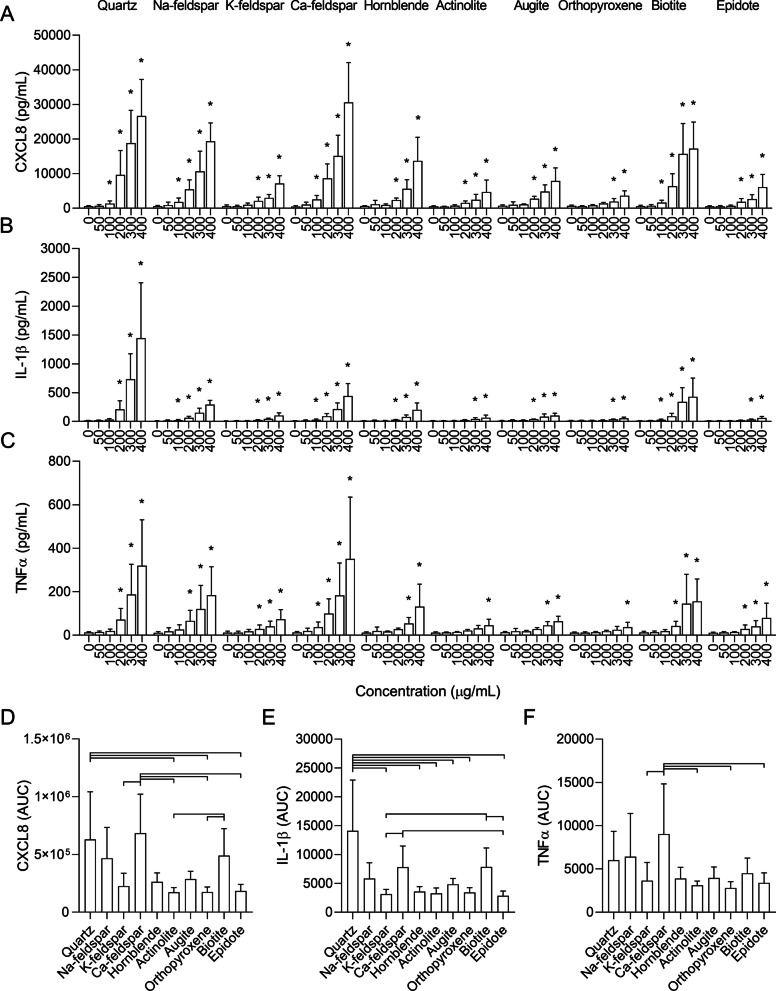
Mineral particles induce release of pro-inflammatory cytokines in THP-1 macrophages. THP-1 macrophages were exposed to 0–400 µg/mL Na-feldspar, K-feldspar, Ca-feldspar, biotite, epidote, hornblende, actinolite, augite, quartz and orthopyroxene for 24 h. The release of CXCL8 (**A** and **D**), IL-1β (**B** and **E**) and TNFα (**C** and **F**) in the cell culture supernatants was measured by ELISA. Results are presented as mean ± SD (*n* = 5). Area under the curve (AUC) values in **D**–**F** were calculated from the values in **A**–**C** for the concentration-range of 0–200 µg/mL. Statistical significance is based on a two-way ANOVA followed by Dunnet’s post-test (**A**–**C**) or a one-way ANOVA followed by Tukey post-test (**D**–**F**). All values were log-transformed prior to statistical analysis to satisfy model assumptions. Asterisks (*) indicate statistically significant difference from the respective control (**A**–**C**), while a capped line indicates statistically significant difference between samples (**D**–**F**)

All particle samples induced a concentration-dependent increase in CXCL8, IL-1β and TNFα in THP-1 macrophages (Fig. 5). However, the particle samples exhibited a different order of potency compared to HBEC3-KT cells. The Ca-feldspar and quartz samples induced the highest CXCL8 responses, followed by Na-feldspar and biotite, and were significantly different compared to control from 100 µg/mL. The K-feldspar, epidote, hornblende and augite samples induced similar levels of CXCL8 that reached statistical significance at 200 µg/mL, while orthopyroxene was the least potent particle sample and did not reach statistical significance before 300 µg/mL. In terms of the magnitude of the response at the highest concentrations, the quartz sample induced the strongest IL-1β response, but the effect was not significant before 200 µg/mL. Conversely, the Na-feldspar, Ca-feldspar and biotite samples induced similar IL-1β responses that were significantly different from control at 100 µg/mL but did not reach as high levels as quartz at the highest concentrations. In comparison, the K-feldspar, hornblende and augite samples induced lower levels of IL-1β that reached statistical significance at 200 µg/mL, while epidote, actinolite and orthopyroxene were the least potent samples, inducing significant levels at 300 µg/mL. Ca-feldspar and quartz were the most potent samples in terms of TNFα secretion and induced similar levels that were statistically significant at 100–200 µg/mL, respectively (Fig. 5C). Na-feldspar and biotite were the second most potent samples and induced significant levels at 200 µg/mL compared with control, while the K-feldspar, epidote and hornblende samples induced similar levels of TNFα that were significant at 200–300 µg/mL (Fig. 5C). Actinolite, augite and orthopyroxene were the least potent inducers of TNFα (Fig. 5C).

A similar order of potency as described above was found when comparing the AUC values of the mineral particle-induced responses, although not all comparisons reached statistical significance (Fig. 5D–F). Based on the results from the cytotoxicity assay (Fig. 3B and D), 0–200 µg/mL was used when calculating the AUC values to avoid underestimating the effect of the most cytotoxic samples. In this concentration-range, the quartz and Ca-feldspar samples were the most potent inducers of CXCL8 and induced similar levels (Fig. 5D). Quartz induced significantly higher levels of CXCL8 than epidote, actinolite and orthopyroxene while the response induced by Ca-feldspar was significantly higher than K-feldspar, epidote, actinolite and orthopyroxene (Fig. 5D). The response induced by the biotite sample was also significantly greater than actinolite and orthopyroxene (Fig. 5D). The Na-feldspar sample induced an intermediate response similar to biotite that was not significantly different compared with the rest of the particle samples (Fig. 5D). For IL-1β, quartz was the most potent particle sample and induced a significantly higher response than K-feldspar, epidote, hornblende, actinolite, augite and orthopyroxene (Fig. 5E). The Ca-feldspar and biotite samples induced similar responses that were significantly higher than K-feldspar and epidote (Fig. 5E). As for CXCL8, Na-feldspar induced an intermediate response that did not reach statistical significance compared to the other particle samples (Fig. 5E). The Ca-feldspar sample also induced significantly higher levels of TNFα than K-feldspar, epidote, actinolite and orthopyroxene (Fig. 5F).

In contrast to the HBEC3-KT cells, no significant correlation was detected between particle size and particle-induced cytokine release in the THP-1 macrophages (Additional file 3: Fig. S2b).

### Hemolysis

**Fig. 6:**
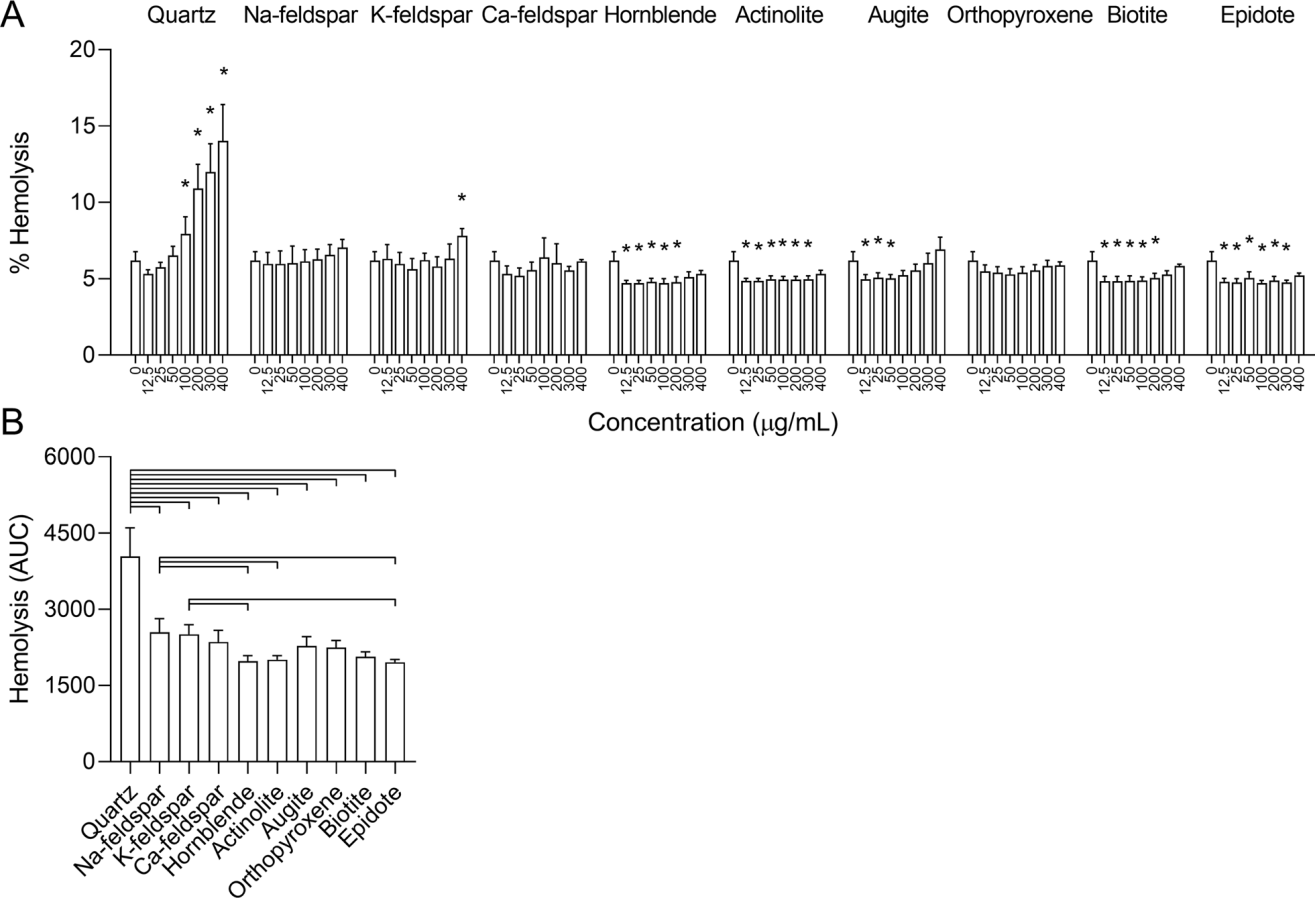
Mineral particle-induced lysis of human erythrocytes. Human erythrocytes harvested from donors were exposed to 0–400 µg/mL Na-feldspar, K-feldspar, Ca-feldspar, biotite, epidote, hornblende, actinolite, augite, quartz and orthopyroxene for 30 min. Free hemoglobin in the supernatant, an indicator of cell lysis, was detected by reading absorbance at 540 nm. Results are presented as mean ± SD (*n* = 3). Area under the curve (AUC) values in B were calculated from the values in A for the concentration-range of 0–400 µg/mL. Statistical significance is based on a two-way ANOVA followed by Dunnet’s post-test (**A**) or a one-way ANOVA followed by Tukey post-test (**B**). All values were log-transformed prior to statistical analysis to satisfy model assumptions. Asterisks (*) indicate statistically significant difference from the respective control (**A**), while a capped line indicates statistically significant difference between particle samples (**B**)

To assess whether differences in membranolytic properties could explain the differences in potency between the mineral particle samples, their ability to lyse human erythrocytes was assessed in the hemolysis assay. In this assay, particle-induced hemolysis serves as a model system for interactions with cellular membranes and has been shown to predict the toxicity of silica particles [30, 31]. Quartz induced the largest degree of hemolysis, culminating at approximately 15% at the highest concentration, and was significantly different from control at 100 µg/mL (Fig. 6A). However, except from a small increase induced by K-feldspar, no significant increases in hemolysis were detected for the rest of the particle samples. Unexpectedly, several of the samples caused small but significant reductions in absorbance compared to controls. As it seems unlikely that mineral particles may have reduced basal hemolysis compared to controls, this may indicate that constituents of the particle samples have interfered with the assay. Thus, it is a possibility that the hemolytic effects of the mineral samples were slightly underestimated by the current procedure. When comparing AUC values, quartz induced significantly higher levels of hemolysis than all the other particle samples (Fig. 6B).

### Association between mineralogical composition and bioactivity

To assess the role of different particle constituents in the observed cytotoxic and pro-inflammatory effects, the results presented in the previous sections were compiled with results of recently published experiments, in which the cytotoxic and pro-inflammatory effects of six stone particle samples of different mineralogical composition and an α-quartz sample were assessed in the same cell models as in the present study [21], and analysed using linear regression models. The content of different minerals were derived from the results of the XRD analyses presented in this study and in Grytting et al. [21] (Additional file 1: Table S1). In the univariable models, the associations between the different cellular responses and individual particle components were assessed using weighted linear regression. In the multivariable models, least absolute shrinkage and selection operator (LASSO) penalized regression was performed to pick out the best fitting variables from multiple linear regression models containing all particle components. The effect estimates reflect percent change in the endpoint for one unit increase in the component. As hemolysis was almost exclusively induced by quartz in this and our previous study [21], further analysis of the statistical associations between mineralogical composition and hemolysis was omitted.

#### Cell viability

**Fig. 7:**
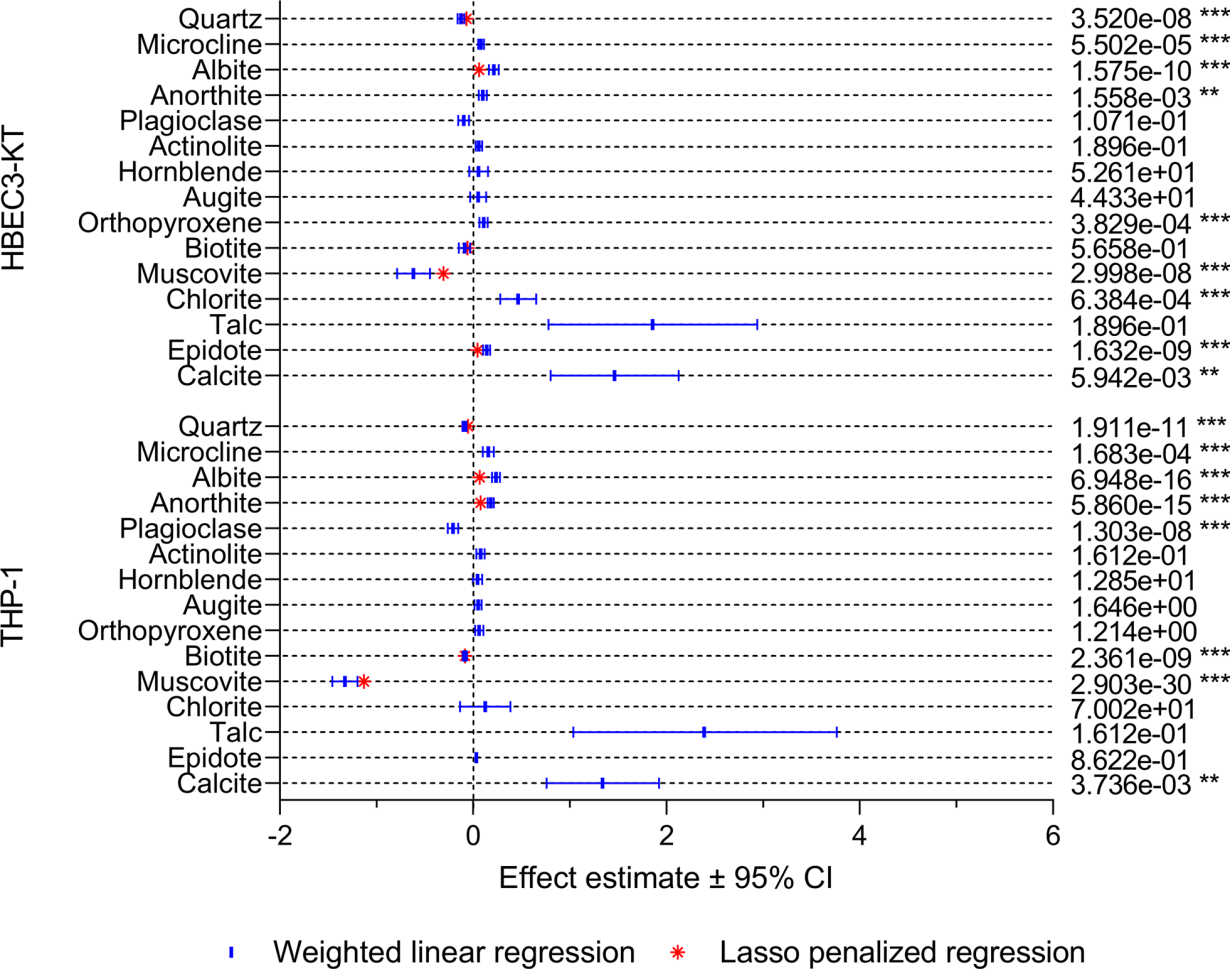
The association between mineralogical composition and particle-induced changes in cell viability in HBEC3-KT cell and THP-1 macrophages. The association between the content of individual mineral components and particle-induced changes in cell viability was assessed using weighted linear regression (blue lines and symbol) and LASSO penalized regression (red symbol). Effect estimates represent percent change in the dependent variable for one unit increase in the independent variable. *p* values < 0.05 was considered statistically significant for the weighted linear regression, while all parameters present in the LASSO penalized models were considered statistically significant. All *p* values were corrected for multiple testing using Bonferroni correction. ***p < 0.001, **p < 0.01, *p < 0.05

The association between particle components and cell viability is presented in Fig. 7. In HBEC3-KT cells, quartz and muscovite were negatively associated with cell viability in both the univariable and multivariable models, while biotite was negatively associated in the multivariable model only. Muscovite had the largest negative effect estimate, followed by quartz and biotite, indicating that mineral dust with high content of these minerals may be more cytotoxic. Microcline, albite, anorthite, epidote, orthopyroxene, calcite and chlorite were positively associated with cell viability in the univariable models, with calcite having the highest effect estimate. Thus, mineral dust with higher levels of these minerals appeared to have lower cytotoxicity. However, only the association between cell viability and albite and epidote remained positive in the multivariable model (Fig. 7).

In THP-1 macrophages, quartz, plagioclase, biotite and muscovite were negatively associated with cell viability in the univariable model, while microcline, albite, anorthite and calcite were positively associated. As for HBEC3-KT, muscovite and calcite had the largest negative and positive effect estimates, respectively. In the multivariable model, quartz, biotite and muscovite were negatively associated with cell viability, with muscovite having the highest effect estimate, while albite and anorthite were positively associated (Fig. 7).

#### Cytokine release

**Fig. 8:**
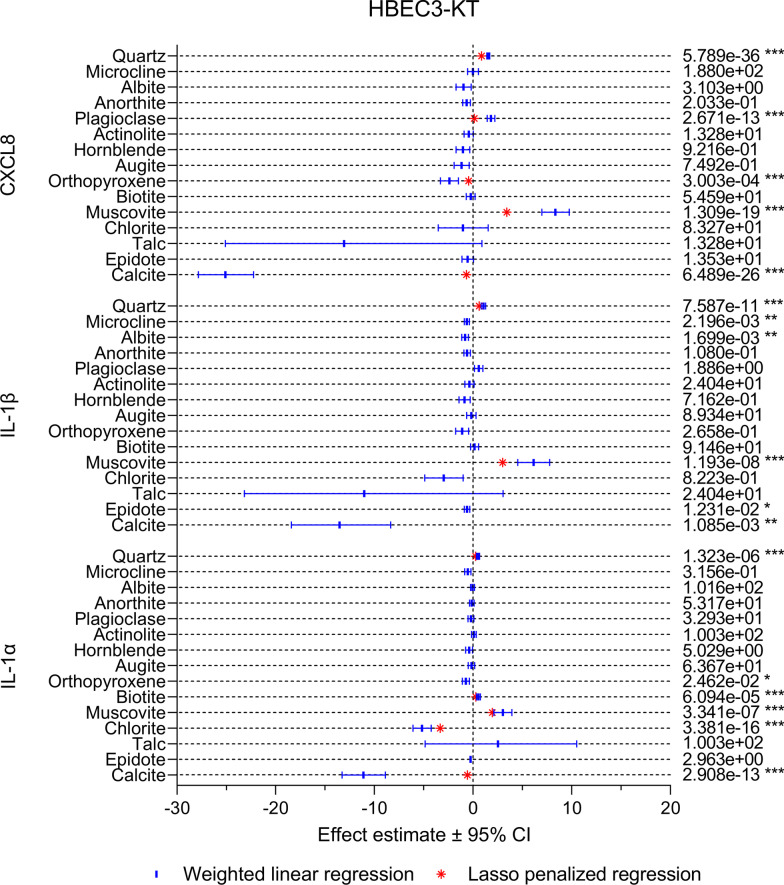
The association between mineralogical composition and particle-induced cytokine release in HBEC3-KT cells. The association between the content of individual mineral components and particle-induced release of CXCL8, IL-1β and IL-1α was assessed using weighted linear regression (blue lines and symbol) and LASSO penalized regression (red symbol). Effect estimates represent percent change in the dependent variable for one unit increase in the independent variable. *p* values < 0.05 was considered statistically significant for the weighted linear regression, while all parameters present in the LASSO penalized model were considered statistically significant. All *p* values were corrected for multiple testing using Bonferroni correction. ***p < 0.001, **p < 0.01, *p < 0.05

In HBEC3-KT cells, a similar pattern was detected for CXCL8, IL-1β and IL-1α (Fig. 8). Quartz and muscovite were the only mineral components that were positively associated with all cytokines in both the univariable and the multivariable model, with muscovite having the largest effects estimate in all cases. Positive associations were also detected between plagioclase and CXCL8 and IL-1β, and between biotite and IL-1α. Calcite was the particle component with the strongest negative association with cytokine release in the univariable models, suggesting that mineral dust with high content of this mineral is less inflammatory. However, the effect estimate for this particle component was severely diminished in the multivariable models for CXCL8 and IL-1α and gone from the IL-1β model. Moreover, calcite was only present in small quantities (< 5%) in a few of the particle samples (Fig. 1 and Additional file 1: Table S1), suggesting that the impact of the calcite content on cellular responses is low. Albite, microcline, epidote, orthopyroxene and chlorite were also negatively associated with one or more of the cytokines in the univariable models, but with lower effects estimates than calcite, suggesting that these mineral components are of less importance. Orthopyroxene and chlorite were also negatively associated with CXCL8 and IL-1α in the multivariable models, respectively.

**Fig. 9:**
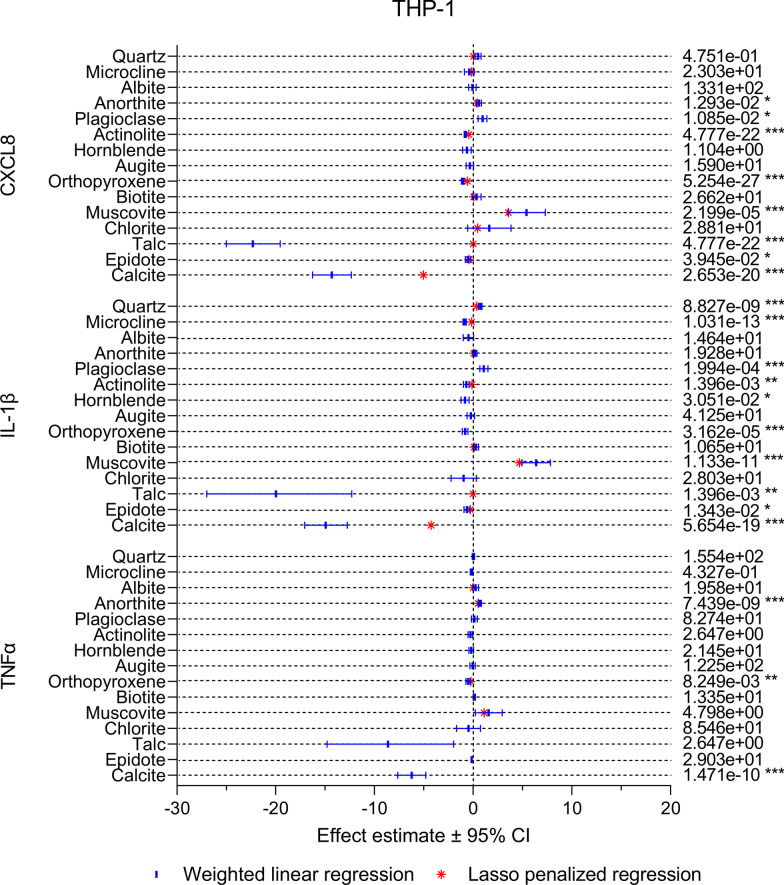
The association between mineralogical composition and particle-induced cytokine release in THP-1 macrophages. The association between the content of individual mineral components and particle-induced release of CXCL8, IL-1β and TNFα was assessed using weighted linear regression (blues lines and symbol) and LASSO penalized regression (red symbol). Effect estimates represent percent change in the dependent variable for one unit increase in the independent variable. *p* values < 0.05 was considered statistically significant for the weighted linear regression, while all parameters present in the LASSO penalized model were considered statistically significant. All *p* values were corrected for multiple testing using Bonferroni correction. ***p < 0.001, **p < 0.01, *p < 0.05

Compared to HBEC3-KT, there were larger differences between the different cytokines in the THP-1 macrophages, as well as between the univariable and multivariable models (Fig. 9). However, similarly to HBEC3-KT, muscovite was positively associated with all cytokines in both models and had the highest effect estimate. Positive associations with lower effects estimates were also detected for plagioclase, anorthite, biotite, albite, chlorite and quartz, depending on the cytokine and model. However, quartz exhibited weaker and less consistent associations with cytokine release in THP-1 macrophages than in HBEC3-KT cells. While talc was the component with the strongest negative association with all cytokines this mineral was only present in small quantities (3%) in the actinolite sample, resulting in wide confidence intervals in the univariable models and low effects estimates in the multivariable models. Thus, talc is likely not a strong predictor of the toxicity of mineral dust. Like in HBEC3-KT, calcite displayed strong negative associations with all cytokines in the univariable models in THP-1 macrophages. The associations remained in the multivariable models for CXCL8 and IL-1β, although diminished, but not for TNFα. Negative associations were also detected for microcline, actinolite, epidote, hornblende and orthopyroxene depending on the cytokine and model, but with lower effect estimates than calcite.

## Discussion

Previous studies show that different stone- and mineral particles differ in their ability to induce cytotoxicity and inflammatory responses [20, 23–25, 27]. However, the reasons for these differences are still unresolved. In the present study, we explored the role of mineralogical composition by assessing the cytotoxic, pro-inflammatory and membranolytic effects of ten mineral particle samples in vitro, and by analysing the statistical association between different mineral components and the bioactivity of mineral dust. Overall, the results suggest that several minerals commonly found in mineral dust may contribute to induce acute inflammatory responses in cells of the human airways. Importantly, the minerals differed in potency, with some minerals inducing effects comparable to quartz, especially with respect to induction of cytokine responses at the lower concentrations.

Previous studies on the toxicity of stone- and mineral particles have provided somewhat conflicting results. The results of older studies from our group indicated that samples with high content of feldspar minerals, either in the form of plagioclase or microcline, were among the least potent inducers of inflammatory cytokines in both human A549 cells, rat Type 2 cells and macrophages, and in rats in vivo [20, 23–27]. Moreover, the content of plagioclase feldspar displayed a strong negative correlation with MIP-2 production in rat Type 2 cells [27]. The low toxicity of feldspar dust is also supported by the results of studies by other groups [32–34]. Conversely, recent findings from our laboratory suggest a more variable biological activity of feldspar-rich particle samples, with samples composed primarily of feldspar inducing cytotoxicity and release of pro-inflammatory cytokines in HBEC3-KT cells and THP-1 macrophages to a similar or greater extent as α-quartz [21]. A possible explanation for the discrepancy between older and recent studies could be the presence of different feldspar minerals. Feldspar is a group of minerals that is further categorized based on the content of the elements K, Na and Ca, often in terms of the respective endmembers. In mineralogy, an endmember is a mineral that represents the end of a mineral series in terms of purity. Plagioclase feldspar consists of solid solutions of Na- and Ca-feldspar, with albite and anorthite forming the Na- and Ca-endmembers, respectively, while solid solutions between albite and the K-feldspar endmember, microcline, are known as alkali feldspars (Additional file 2: Fig. S1). The anorthosite assessed in our previous study consisted primarily of plagioclase, while the less potent rhomb porphyry contained high amounts of microcline in addition to plagioclase [21], suggesting that the differences in potency between these particle samples may be due to the presence of different feldspar minerals. In support of this, the results of the present study showed that the K-feldspar sample, which consisted primarily of microcline, exhibited a lower pro-inflammatory potential in THP-1 macrophages than the Na-feldspar and Ca-feldspar samples, which consisted primarily of albite and anorthite. Moreover, the regression analyses revealed negative associations between microcline content and release of CXCL8 and IL-1β in THP-1, while anorthite was positively associated with release of all cytokines. In addition, the content of plagioclase, which consists of both Na- and Ca-feldspar, was associated with increased CXCL8 and IL-1β release in the univariable models only. Conversely, no difference in inflammatory potential was detected between the feldspar samples in HBEC3-KT, although plagioclase content was associated with increased CXCL8 secretion, and microcline and albite were negatively associated with IL-1β secretion in the univariable model only. Furthermore, intra-tracheal instillation of anorthosite dust, composed primarily of anorthite, produced no effect on markers of lung toxicity and inflammation in rats [33, 34]. However, Damby et al. [35] reported increased release of IL-1β in murine macrophages after exposure to labradorite, an intermediate plagioclase containing 50–70% anorthite, although the effect was modest compared with cristobolite silica. Taken together, the results suggest that the ability of feldspar to induce pro-inflammatory responses may differ between feldspar minerals, with anorthite and albite having higher potency than microcline. However, the effect appears to be dependent on the model system. Thus, the full extent of the contribution of feldspar minerals to the toxicity of mineral dust is still inconclusive.

In addition to feldspar, the feldspar-rich stone particle samples used in previous studies contained varying amounts of other minerals, such as chlorite, calcite, biotite, amphibole, mica, pyroxene, hornblende, augite, epidote, biotite, muscovite and quartz [21, 23, 27]. Interestingly, muscovite was the only particle component that was consistently associated with all endpoints in the regression analyses in the present study, suggesting that particle samples with high content of this mineral may be more cytotoxic and pro-inflammatory. Muscovite belongs to the mica group of the phyllosilicates, a group of minerals characterized by their sheet-like morphology. The muscovite was primarily present in the anorthosite sample studied in Grytting et al. [21] and likely occurs as sericite, a fine-grained mica similar to muscovite, illite and paragonite that forms through degradation of feldspar minerals [36]. Biotite is also a member of the mica group of phyllosilicates, and shares similarities with muscovite, but with higher iron content. Biotite was the most cytotoxic mineral tested, in both HBEC3-KT cells and THP-1 macrophages, and induced cytokines at the lowest concentrations in the HBEC3-KT cells. However, only weak associations were detected with cytotoxicity in both cell types and with CXCL8 and IL-1β in THP-1 in the regression analyses. This is likely because, apart from the pure biotite sample, the mineral was only present in small amounts in the other particle samples included in the analyses (Fig. 1 and Additional file 1: Table S1). Several phyllosilicates, including the mica minerals muscovite and phlogopite, are of great commercial interest and are used in a diverse range of applications, such as paper, plastics, rubber, ceramics, building materials, electronics and cosmetics [2]. As reviewed by Skulberg et al. [6] studies suggest that occupational exposure through mining, milling or packing mica may increase the risk of developing pneumoconiosis. However, the development of pneumoconiosis due to exposure to mica or other phyllosilicates seems to require prolonged exposure to very high concentrations of dust, and the disease-relationship is often confounded by exposure to other occupational hazards, such as quartz and asbestos [6, 37]. Nevertheless, several case reports indicate that high exposure to mica dust may cause pneumoconiosis distinct from silicosis in the absence of other occupational hazards [38–44]. While the number of experimental studies is scarce, toxicity from exposure to mica has been reported. Work by Sahu et al. showed that intra-tracheal instillation of mica dust can cause pulmonary inflammation and mild fibrogenic responses in rats and mice, although the authors noted that the lesions in mice were different from those occurring in humans [45, 46]. Similarly, Rosmanith et al. found that intratracheal instillation of muscovite caused fibrogenic reactions in rats, with the finest dust fractions causing the greatest response [47]. An in vitro study assessing the toxicity of differently prepared samples of phlogopite found that the mineral induced cytotoxicity and cytokine secretion in a concentration-dependent manner in mouse RAW 264.7 macrophages to a similar or greater extent than quartz [48]. Phlogopite is also known as magnesium mica and constitutes the magnesium endmember of the biotite series of minerals. Given the association between mica and particle toxicity in the present study, it is tempting to speculate that the sheet-like structure of mica may represent another characteristic contributing to particle toxicity, similar to the well-defined Fibre Pathogenicity Paradigm derived from studies of asbestos and nanofibers [49]. In line with this notion, Holopainen et al. [48] proposed that the presence of larger intact sheets of phlogopite was the reason for the increased toxicity of their water-elutriated sample [48]. A thin mineral sheet may be expected to have a larger surface area than a spherical or irregular particle of the same diameter, which may account for the increased toxicity. Regrettably, the limited amounts of available sample material precluded the possibility of measuring the surface area of the mineral samples in the present study. Taken together, our present findings lend support to previous studies and suggest that the health effects of mica minerals may warrant further attention.

Quartz is a known pathogenic mineral that can cause silicosis and lung cancer when inhaled [16, 18]. In the present study, the pure quartz sample was among the most cytotoxic and pro-inflammatory particle samples in both cell types. This is in line with the results from Grytting et al. [21], which suggested that α-quartz and stone particle samples composed primarily of quartz have high bioactivity. In the regression analysis, quartz content was associated with increased cytotoxicity in both cell models. Positive associations were also detected between quartz content and CXCL8, IL-1β and IL-1α in HBEC3-KT cells and with IL-1β and CXCL8 in THP-1 macrophages. However, the effect estimate for CXCL8 release in THP-1 macrophages was relatively low and no association was detected between quartz content and TNFα. This could possibly be explained by the low bioactivity of some quartz-rich particle samples assessed in Grytting et al. [21] that were included in the regression analyses in the present study. Moreover, the effects of the quartz sample in the present study were mainly observed at the highest concentrations, which were excluded from the cytokine AUC calculations, and hence also the regression analyses, to minimize the impact from cytotoxicity. At the lower concentrations, several other mineral samples appeared to be equally or even more potent. Nevertheless, these findings underscore that even though quartz particles can induce strong inflammatory responses and cytotoxicity in HBEC3-KT cell and THP-1 macrophages, quartz content in itself may not fully predict the toxicity of mineral dusts.

It is important to keep in mind that studies on quartz suggest that even a single mineral may vary considerably in bioreactivity. As stated by Donaldson and Borm [50] “*the hazard posed by quartz is not a constant entity, but one that may vary dramatically depending on the origin of the silica sample or its contact with other chemicals/minerals within its complex constitution”.* If the toxicity of quartz depends on the sample origin or specific particle properties, it seems reasonable to assume that similar factors may affect the toxicity of other mineral particles. Thus, the statement in particle toxicology of the quartz hazard being “*a variable entity*” likely extends to most other biologically reactive minerals. Any such variability in toxicity of a given mineral would necessarily affect the statistical analysis of associations between mineralogical composition and biological effects.

While the present study supports the notion that minerals other than quartz can induce acute inflammatory responses in macrophages and pulmonary epithelial cells, the mechanisms remain elusive. The toxicity of quartz is hypothesized to be caused by the reactive surface of fractured particles, which causes destabilisation of the lysosomal membrane and the subsequent activation of the nucleotide-binding oligomerization domain (NOD)-like receptor containing pyrin domain 3 (NLRP3) inflammasome through leakage of lysosomal content [51]. The erythrocyte membrane has been used as a model for cellular membranes, and particle-induced hemolysis has been shown to correlate well with the toxicity of quartz particles in experimental studies [30, 31]. However, with the exception of quartz, the mineral samples in the present study induced a negligible degree of hemolysis, suggesting that the particles exert their effects through a different mechanism. The reactivity of quartz stems from moieties such as nearly free silanols, which are generated at conchoidal fractures at the particle surface [52]. However, quartz is the only mineral assessed in the present study that displays this cleavage pattern. Thus, the absence of conchoidal fractures can possibly explain the poor membranolytic potential of the other mineral samples, as well as the stone particle samples assessed in Grytting et al. [21]. Nevertheless, previous studies from our group suggested that stone particle samples without quartz and with low hemolytic potential were still able to activate the NLRP3 inflammasome pathway in THP-1 macrophages, suggesting that this may also be the case for the minerals assessed in the present study [21].

Several of the mineral samples used in the present study contain metals that have been reported to induce ROS, inflammation and cytotoxicity in pulmonary cells, suggesting a potential role in the particle-induced effects reported in the present study [53–57]. Metals present in both mineral particles and ambient PM can be soluble in physiological solutions, and the dissolution may increase in low pH environments such as found in the lysosomes [26, 58–60], suggesting that the metals may be available for interaction with cells. Several of the particle samples assessed in the present study contained considerable amounts of iron, a transition metal known to contribute to ROS formation and oxidative stress through Fenton and Haber–Weiss reactions. Biotite contained the largest amount of iron of all the particle samples, consisting of 25% Fe_2_O_3_, followed by orthopyroxene at 17%, hornblende and epidote at 13–14%, and actinolite and augite at 7–8%. Studies suggest that fractured stone- and mineral particles can generate ROS such as hydroxyl radicals and hydrogen peroxide in cell-free solutions [26, 61–63], and that the oxidative potential is greater for mineral particles with high iron-content [61, 62, 64, 65]. Moreover, work by Costa et al. suggests that biotite has a high capacity to generate ROS [64, 65]. However, the correlation between iron content and particle-induced inflammation and cytotoxicity is not clear-cut, as the biotite, epidote, hornblende and orthopyroxene samples assessed in the present study all contained large amounts of iron but varied greatly in potency. In line with this, Øvrevik et al. [26] did not detect any correlation between total or soluble iron content, ROS generation and the ability of mylonite, gabbro, basalt, feldspar, jasper, forsterite, anti-spinell and quartz particles to induce inflammatory cytokines and cytotoxicity in A549 cells. Similarly, Hetland et al. [22] detected no correlation between the ability to generate cellular- and acellular ROS and cytokine secretion induced by mylonite, gabbro, basalt and feldspar particles. Interestingly, a report from the Geological Survey of Norway shows that biotite is more soluble in hydrochloric and nitric acid compared to several other minerals, including epidote [66], suggesting that the iron in this mineral may be more available for interaction with cells. In line with this, experiments with chrysotile, crocidolite and erionite fibers show that the dissolution of metals in simulated biological fluids depends on the solubility of the minerals and is not necessarily reflected by total metal content [58]. Likewise, a discrepancy between the total and soluble iron in mylonite, gabbro, feldspar, jasper, forsterite, anti-spinell and quartz particles has been reported [26]. As no attempt was made in the present study to assess the bioavailability of the metals in the different particle samples in physiologically relevant solutions, their role in the observed particle-induced effects is hard to discern. Nevertheless, the present study cannot exclude the possibility that metals and ROS can contribute to the toxicity of some of the mineral samples such as biotite.

It should be noted that there were marked differences in particle size between some of the samples used in the present study. In particular, there was a large discrepancy between the hornblende and biotite samples, which contained the most small- and large-sized particles, respectively. The larger particle size in the biotite sample is likely due to the sheet-like morphology of this mineral, which causes the particles to settle more slowly in water during extraction of the < 10 µm fraction of particles. However, the reason for the smaller size of the hornblende sample is uncertain. A significant association was detected between particle size and particle-induced release of IL-1α and IL-1β in the HBEC3-KT cells, suggesting that some of the differences between the mineral samples may be due to particle size rather than mineral-intrinsic properties. However, this result is in disagreement with the common notion of smaller particles being more potent than larger particles on an equal mass basis, due to larger surface area per mass [67–70]. Thus, we suspect that the apparent association between particle size and cytokine release observed in the present study is by chance rather than evidence of particle size as an underlying cause. This is also supported by the lack of a similar association in the THP-1 macrophages. However, it should be considered that since particle size may influence the potency of particles of the same composition, the potency of samples with smaller-sized particles may have been somewhat overestimated in the current study. Conversely, the potency of samples with a larger particle size may have been underestimated. A possible exception is the biotite sample, as a previous study of the pro-inflammatory effects of phlogopite mica reported that the sample with the largest and most intact sheets was the most potent [48]. However, as we have not compared the effect of differently sized samples of the same mineral, firm conclusions regarding the impact of particle size for the different mineral samples cannot be drawn.

## Conclusions

The results of the present study provide further evidence that minerals other than quartz can induce inflammatory responses in cells of the human airways. All tested minerals induced biological effects to some extent, but their potency differed considerably. In contrast to previous studies showing that feldspar minerals have low potency, the present study suggests a more variable bioactivity of feldspar minerals, with anorthite and albite being more potent than microcline. The high pro-inflammatory potential of the biotite sample and the consistent association between bioactivity and muscovite content in the regression analyses is of particular interest, as it suggests that dust with high content of sheet-like mica minerals may be more hazardous. Whether a sheet-like structure could represent a property affecting particle toxicity, for instance by providing a larger surface-to-mass ratio, is an intriguing question that may warrant further attention.

## Materials and methods

### Particle preparation and characterization

#### Generation of particles < 10 µm

Samples of Na-feldspar, K-feldspar, Ca-feldspar, biotite, epidote, hornblende, actinolite, augite, quartz and orthopyroxene were purchased or collected by the Geological Survey of Norway in the form of hand specimens. After visually inspecting the material and removing impurities, the samples were crushed in a jaw crusher equipped with low-Cr steel plates. The Na-feldspar, K-feldspar, Ca-feldspar and biotite samples were already crushed upon delivery. Next, the crushed samples of Na-feldspar, K-feldspar, Ca-feldspar, epidote, hornblende, actinolite, augite, quartz and orthopyroxene were ground in a vibrating agate disc mill. The biotite sample was ground in an agate ball mill as pure mica particles would lubricate the chamber of the agate disc mill and produce inadequate results. The difference in preparation method is not expected to influence the potency of the resulting particles. Finally, the fraction of particles < 10 µm in diameter were separated by gravity settling in deionized water and extracted as described in Grytting et al. [21].

#### Geochemical and mineralogical characterization

The characterization of the particle samples was conducted using X-ray diffraction (XRD) and X-ray fluorescence (XRF) spectroscopy as described previously [21]. The geochemical composition of the samples was analysed with a PANalytical Axios sequential wavelength-dispersive X-ray spectrometer operating with a 4 kW Rh-tube. Mineralogical analyses were carried out with a Bruker D8 Advance diffractometer (Cu Kα radiation in 3–75° 2θ range). Minerals were identified using automatic/manual peak search & match function with Bruker's Diffraction EVA V4.1 software using Crystallographic Open Database and PDF4 Minerals database from the International Centre of Diffraction Data and quantified using Rietveld modelling in TOPAS 5 software.

#### Particle size distribution

The distribution of particle size was determined by a Beckman Coulter LS13320 Laser Particle size analyser as described previously [21]. Sample suspensions were prepared in water with 5% sodium pyrophosphate and sonicated with MSE ultrasonic disintegrator at amplitude 14 for 5 min. The biotite, hornblende, orthopyroxene samples were analysed from powder, but otherwise treated in the same way as the suspension samples.

#### Endotoxin contamination

The concentrations of bacterial endotoxin in the particle samples were measured using the Pierce™ Chromogenic Endotoxin Quant Kit (ThermoFisher Scientific, Waltham, MA, USA) with minor modifications noted in Grytting et al. [21].

### Cell cultures and exposure

#### THP-1

THP-1 cells were cultured in RPMI 1640 cell culture medium with L-glutamine (Gibco, Thermo Fischer Scientific, Waltham, MA, USA), supplemented with sodium pyruvate (Sigma-Aldrich, St. Louis, MO, USA), hepes (Sigma-Aldrich, St. Louis, MO, USA), gentamicin (Gibco, Thermo Fischer Scientific, Waltham, MA, USA) and 10% foetal calf serum (FCS; Biochrom, Berlin, Germany). Cells were maintained at 37 °C in a humidified atmosphere containing 5% CO_2_ and passaged every 2–3 days to maintain a density of approximately 5 × 10^5^ cells/mL. For experimental procedures, the cells were seeded in 6-well Corning^®^ Costar^®^ cell culture plates (Merck, Darmstadt, Germany) at a density of 500 000 cells/mL in 2 mL cell culture medium, and differentiated into macrophage-like cells by treatment with 64 nM phorbol myristate acetate (PMA; Merck, Darmstadt, Germany) for 48 h at 37 °C in an atmosphere containing 5% CO_2_. Following differentiation to THP-1 macrophages, the medium was removed and the cells washed once with Dulbecco’s phosphate buffered saline (PBS) before replacing the medium with serum-free RPMI.

#### HBEC3-KT

Human bronchial epithelial cells (HBEC3-KT) were cultured in LHC-9 medium (Lonza, Basel, Switzerland) in collagen-coated T75 flasks and maintained at 37 °C in a humidified atmosphere containing 5% CO_2_. Twice every week the cell culture was loosened from the flask and passaged to maintain proper conditions. Prior to the experiments, cells were seeded on collagen-coated 6-well cell culture plates at a concentration of 2.2 × 10^5^ cells per well in 1 mL LHC-9 medium. After 24 h, the LHC-9 medium was replaced and the cells incubated for an additional 24 h. Then, 24 h prior to exposure, each well was washed with 1 mL PBS before adding 1 mL of serum-free DMEM (Gibco, Thermo Fischer Scientific, Waltham, MA, USA) supplemented with penicillin–streptomycin (Lonza, Basel, Switzerland), ampicillin (New York, NY, USA) and amphotericin B (Sigma, St. Louis, MO, USA).

#### Exposure regime and preparation of particle stock solutions

Particle stock solutions were prepared in serum-free cell culture medium. To ensure even suspension of the particles, the stock solutions were sonicated for 5 min on ice using a Vibra-Cell™ probe sonicator (Sonics & Materials Inc., Newtown, CT, USA). The primary particle stock solutions had a concentration of 2 mg/mL and were further diluted in the wells to yield the final concentrations of 50, 100, 200, 300 and 400 µg/mL. HBEC3-KT cells and THP-1 macrophages were exposed in 1 mL serum-free DMEM and RPMI, respectively. Following exposure, the cells were incubated for 24 h at 37 °C in an atmosphere containing 5% CO_2_. After 24 h, the cell culture medium was transferred to 1.5 mL Eppendorf tubes and centrifuged at 290 × g for 10 min to remove cells and debris. The supernatant was then transferred to new Eppendorf tubes and centrifuged for 10 min at 1200 × g to remove particles. Following centrifugation, the supernatant was transferred to new Eppendorf tubes and stored at − 80 °C awaiting analysis.

### Enzyme-linked immunosorbent assay

The concentrations of cytokines in the cell culture supernatants were measured using Cytoset (TNFα and CXCL8, Invitrogen by Thermo Fischer Scientific and Novex by Life Technologies) or Duoset (IL-1α and IL-1β, R&D systems, Minneapolis, MN, USA) enzyme-linked immunosorbent assay (ELISA) kits, both applied according to the manufacturer’s instructions. ELISA was performed in Nunc Maxisorb plates (Thermo Scientific, Waltham, MA, USA). Absorbance was measured using a Tecan Sunrise plate reader (Tecan, Männedorf, Switzerland).

### Cytotoxicity

Cytotoxicity was assessed by the alamarBlue assay (Invitrogen by Thermo Fischer Scientific, Waltham, MA, USA) according to the manufacturer’s instructions. The cells were incubated for 60 min with alamarBlue solution diluted 1:10 in cell culture medium at 37 °C in an atmosphere containing 5% CO_2_. Fluorescence was measured using a Clariostar plate reader (BMG LABTECH, Ortenberg, Germany).

### Hemolysis assay

The hemolysis assay was adapted from Pavan et al. [71] and performed as described in Grytting et al. [21]. Informed consent was obtained from all volunteers (*n* = 3) and ethical approval was obtained from the Norwegian Regional Committees for Medical and Health Research Ethics (REC,2015/1322). Briefly, donor blood was sampled in 10 mL vacutainers containing K_2_EDTA (BD, Franklin Lakes, NJ, USA). Next, the blood samples were separated by centrifugation and the erythrocytes washed and diluted in 0.9% saline. The erythrocyte suspension was then incubated with 0–400 µg/mL particles for 30 min in a 96 well cell culture plate. After incubation, the plate was centrifuged to remove particles and intact erythrocytes. The supernatant was then transferred to a new 96 well plate and absorbance measured at 540 nm using a Sunrise plate reader (Tecan, Männedorf, Switzerland) to detect free hemoglobin. Results are presented at a percentage of the positive control (0.1% triton X-100; Thermofischer, Waltham, MA, USA).

### Statistical analyses

Statistical analyses were performed using Graphpad Prism 8 (version 8.0.1) or R (version 3.5.0) software.

#### The effects of particle exposure

Statistically significant differences between particle-exposed cells and control were determined using two-way ANOVA followed by a Dunnett’s post-test. For between-particle comparisons the area under the curve (AUC) was calculated for the concentration–response data for each experiment using the trapezoid rule. AUC values were then compared using one-way ANOVA and Tukey post-test. Cell viability data were normalized to reflect percent viability before calculating the AUC values to account for differences in baseline values. Based on the evaluation of residual- and QQ plots, several datasets were log-transformed before analysis due to non-normality and heteroscedasticity. The association between the different cellular endpoints and particle size and the content of endotoxin was assessed by Pearson correlation, using the mean AUC value calculated for each particle sample. In all cases, p values below 0.05 were considered statistically significant.

#### The association between mineralogical composition and bioactivity

The association between mineralogical composition and bioactivity was assessed using linear regression models. QQ plots and residual plots were evaluated to assess model assumptions. The content of the different mineral components detected in the XRD analyses (Additional file 1: Table S1) was used as the independent variable, while the AUC values for cytokine responses and cell viability presented in Figs. 3, 4 and 5 in this paper and Figs. 2, 3 and 4 in Grytting et al. [21] were used as the dependent variable. In the univariable models, the associations between the content of individual mineral components and the AUC values for the different cellular endpoints were assessed using weighted linear regression models. Weights were applied to each individual model according to Eq. 1 due to violations of the assumption of homoscedasticity.

1 $$Weights = \left| \frac{1}{residual} \right|$$

In the multivariable analyses, LASSO penalization was applied to multiple linear regression models containing all the different mineral components to pick out the best fitting parameters predicting each cellular endpoint. LASSO penalization performs both variable selection and regularization by creating multiple possible regression model fits, which are then evaluated via a cross validation procedure [72]. In the present study, leave-one-out cross validation was used to evaluate the regression model fits. In the case of both the univariable and multivariable models, the dependent variables were log-transformed due to non-normality. Consequently, the regression coefficients were transformed according to Eq. 2 to reflect percent change in the dependent variable due to a one-unit change in the independent variable.

2 $$\% {\Delta }y = 100*\left( {e^{\beta } - 1} \right)$$

Bonferroni correction was used to correct for multiple testing in the univariable models and corrected *p* values below 0.05 were considered statistically significant. For the multivariable models, parameters present in the selected model (i.e., selected via LASSO) were considered statistically significant.

## Supplementary Information

**Additional file 1:** Table S1 and Table S2.

**Additional file 2: Fig. S1.** Feldspar phase diagram. Ternary phase diagram of the feldspars (By Muskid, CC BY-SA 4.0, https://commons.wikimedia.org/w/index.php?curid=46491727).

**Additional file 3: Fig. S2.** The association between particle size and particle-induced cytokine release. The association between particle-induced cytokine release in HBEC3-KT cells **A** and THP-1 macrophages **B** was assessed using linear regression. Mean area under the curve (AUC) values for cytokine release were derived from Figs. 4 and 5, while the particle diameter at 50% cumulative volume for each of the mineral samples was derived from Fig. 2.

## Data Availability

The datasets used in the present study are available from the corresponding author upon reasonable request.

## Abbreviations

AUC: Area under the curve
CXCL: Chemokine CXC-motif ligand
DMSO: Dimethyl sulfoxide
ELISA: Enzyme-linked immunosorbent assay
HBEC3-KT: Human bronchial epithelial cells
IL: Interleukin
LAL: Limulus amebocyte lysate
LASSO: Least absolute shrinkage and selection operator
NLRP3: Nucleotide-binding oligomerization domain (NOD)-like receptor containing pyrin domain 3
ASC: Apoptosis-associated speck-like protein containing a CARD
PM: Particulate matter
PMA: Phorbol myristate acetate
ROS: Reactive oxygen species
TNF: Tumor necrosis factor
XRD: X-ray diffraction
XRF: X-ray fluorescence
