# Supplementary material for: The importance of mineralogical composition for the cytotoxic and pro-inflammatory effects of mineral dust
Source: Part Fibre Toxicol. 2022 Jul 6;19:46. doi: 10.1186/s12989-022-00486-7 (PMC9261052; doi:10.1186/s12989-022-00486-7)
Supplement: Supplementary file 1 — Additional file 1: Table S1 and Table S2. [file 12989_2022_486_MOESM1_ESM.docx]

Table S1. Mineralogical composition of the stone- and mineral particle samples. The content of quartz (Quar), microcline (Micr), albite (Albi), anorthite (Anor), plagioclase (Plag), actinolite (Acti), hornblende (Horn), augite (Augi), orthopyroxene (Orpx), biotite (Biot), muscovite (Musc), chlorite (Chlor), talc, epidote (Epid) and calcite (Calc) was determined using X-ray diffraction analysis and is presented as percentages. ^(1)^ The mineral composition of the quartzite, anorthosite, rhomb porphyry, dacite, quartz diorite, hornfels and α-quartz samples are already published in Grytting et al. (2021).

|  | Tectosilicates | | | | |  | Inosilicates | | | |  | Phyllosilicates | | | | Sorosilicates | Carbonates |
| --- | --- | --- | --- | --- | --- | --- | --- | --- | --- | --- | --- | --- | --- | --- | --- | --- | --- |
| Sample | Quar | Micr | Albi | Anor | Plag |  | Acti | Horn | Augi | Orpx |  | Biot | Musc | Chlo | Talc | Epid | Calc |
| Quartz | 100 | 0 | 0 | 0 | 0 |  | 0 | 0 | 0 | 0 |  | 0 | 0 | 0 | 0 | 0 | 0 |
| Na-feldspar | 7 | 15 | 69 | 9 | 0 |  | 0 | 0 | 0 | 0 |  | 0 | 0 | 0 | 0 | 0 | 0 |
| K-feldspar | 3 | 71 | 26 | 0 | 0 |  | 0 | 0 | 0 | 0 |  | 0 | 0 | 0 | 0 | 0 | 0 |
| Ca-feldspar | 1 | 0 | 0 | 99 | 0 |  | 0 | 0 | 0 | 0 |  | 0 | 0 | 0 | 0 | 0 | 0 |
| Hornblende | 1 | 0 | 9 | 0 | 0 |  | 0 | 71 | 0 | 0 |  | 9 | 0 | 5 | 0 | 0 | 5 |
| Actinolite | 0 | 0 | 0 | 0 | 0 |  | 95 | 0 | 0 | 0 |  | 0 | 0 | 0 | 3 | 0 | 2 |
| Augite | 10 | 0 | 0 | 0 | 0 |  | 0 | 14 | 73 | 0 |  | 0 | 0 | 0 | 0 | 0 | 3 |
| Orthopyroxene | 1 | 0 | 0 | 15 | 0 |  | 0 | 0 | 0 | 71 |  | 0 | 0 | 9 | 0 | 0 | 4 |
| Biotite | 0 | 0 | 0 | 0 | 0 |  | 0 | 0 | 0 | 0 |  | 100 | 0 | 0 | 0 | 0 | 0 |
| Epidote | 0 | 0 | 0 | 0 | 0 |  | 0 | 0 | 0 | 0 |  | 0 | 0 | 0 | 0 | 100 | 0 |
| Quartzite ^(1)^ | 87 | 1 | 0 | 0 | 1 |  | 0 | 0 | 0 | 0 |  | 0 | 9 | 0 | 0 | 0 | 0 |
| Anorthosite ^(1)^ | 0 | 0 | 0 | 0 | 61 |  | 0 | 0 | 0 | 0 |  | 0 | 23 | 3 | 0 | 13 | 0 |
| Rhomb porphyry ^(1)^ | 4 | 33 | 0 | 0 | 46 |  | 0 | 7 | 0 | 0 |  | 0 | 2 | 6 | 0 | 0 | 2 |
| Dacite ^(1)^ | 24 | 0 | 0 | 0 | 50 |  | 0 | 0 | 2 | 0 |  | 0 | 11 | 4 | 0 | 9 | 0 |
| Quartz diorite ^(1)^ | 25 | 12 | 0 | 0 | 30 |  | 0 | 0 | 0 | 0 |  | 0 | 4 | 15 | 0 | 14 | 0 |
| Hornfels ^(1)^ | 19 | 34 | 0 | 0 | 33 |  | 0 | 0 | 0 | 0 |  | 4 | 0 | 8 | 0 | 0 | 2 |
| α-quartz ^(1)^  (Min-U-Sil 5) | 100 | 0 | 0 | 0 | 0 |  | 0 | 0 | 0 | 0 |  | 0 | 0 | 0 | 0 | 0 | 0 |

Table S2. The chemical formula of the mineral components.

| Mineral | Chemical formula |
| --- | --- |
| Quartz | SiO_2_ |
| Microcline | KAlSi_3_O_8_ |
| Albite | NaAlSi_3_O_8_ |
| Anorthite | CaAl_2_Si_2_O_8_ |
| Plagioclase | NaAlSi_3_O_8_ - CaAl_2_Si_2_O_8_ |
| Actinolite | Ca_2_(Mg_4.5-2.5_Fe^2+^_0.5-2.5_)Si_8_O_22_(OH)_2_ |
| Hornblende | Ca_2_(Mg,Fe,Al)_5_(Al,Si)_8_O_22_(OH)_2_ |
| Augite | (Ca,Na)(Mg,Fe,Al,Ti)(Si,Al)_2_O_6_ |
| Orthopyroxene | (Mg,Fe)_2_Si_2_O_6_ |
| Biotite | K(Mg,Fe,Al)_3_Si_3_O_10_(OH,F)_2_ |
| Muscovite | KAl_2_(AlSi_3_O_10_)(F,OH)_2_ |
| Chlorite | (Mg,Fe)_3_(Si,Al)_4_O_10_(OH)_2_·(Mg,Fe)_3_(OH)_6_ |
| Talc | Mg_3_Si_4_O_10_(OH)_2_ |
| Epidote | Ca_2_Al_2_(Fe^3+^,^-^Al)(SiO_4_)(Si_2_O_7_)O(OH) |
| Calcite | CaCO_3_ |
