# Supplementary figures and images for: The importance of mineralogical composition for the cytotoxic and pro-inflammatory effects of mineral dust
Source: Part Fibre Toxicol. 2022 Jul 6;19:46. doi: 10.1186/s12989-022-00486-7 (PMC9261052; doi:10.1186/s12989-022-00486-7)

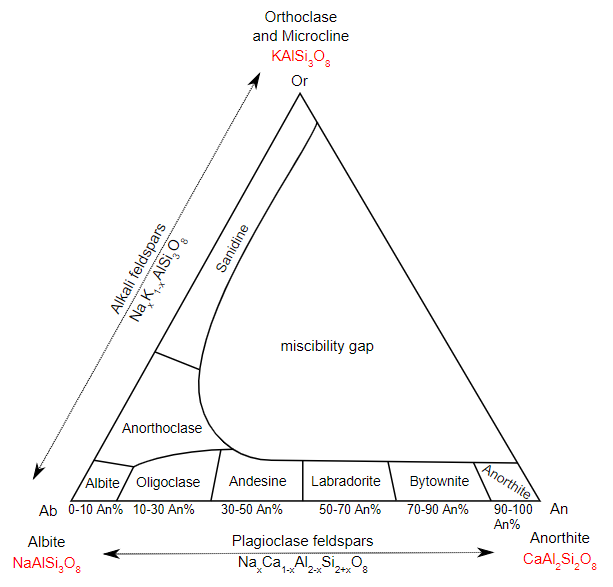

Supplement: Supplementary file 2 — Additional file 2: Fig. S1. Feldspar phase diagram. Ternary phase diagram of the feldspars (By Muskid, CC BY-SA 4.0, https://commons.wikimedia.org/w/index.php?curid=46491727). [file 12989_2022_486_MOESM2_ESM.png]

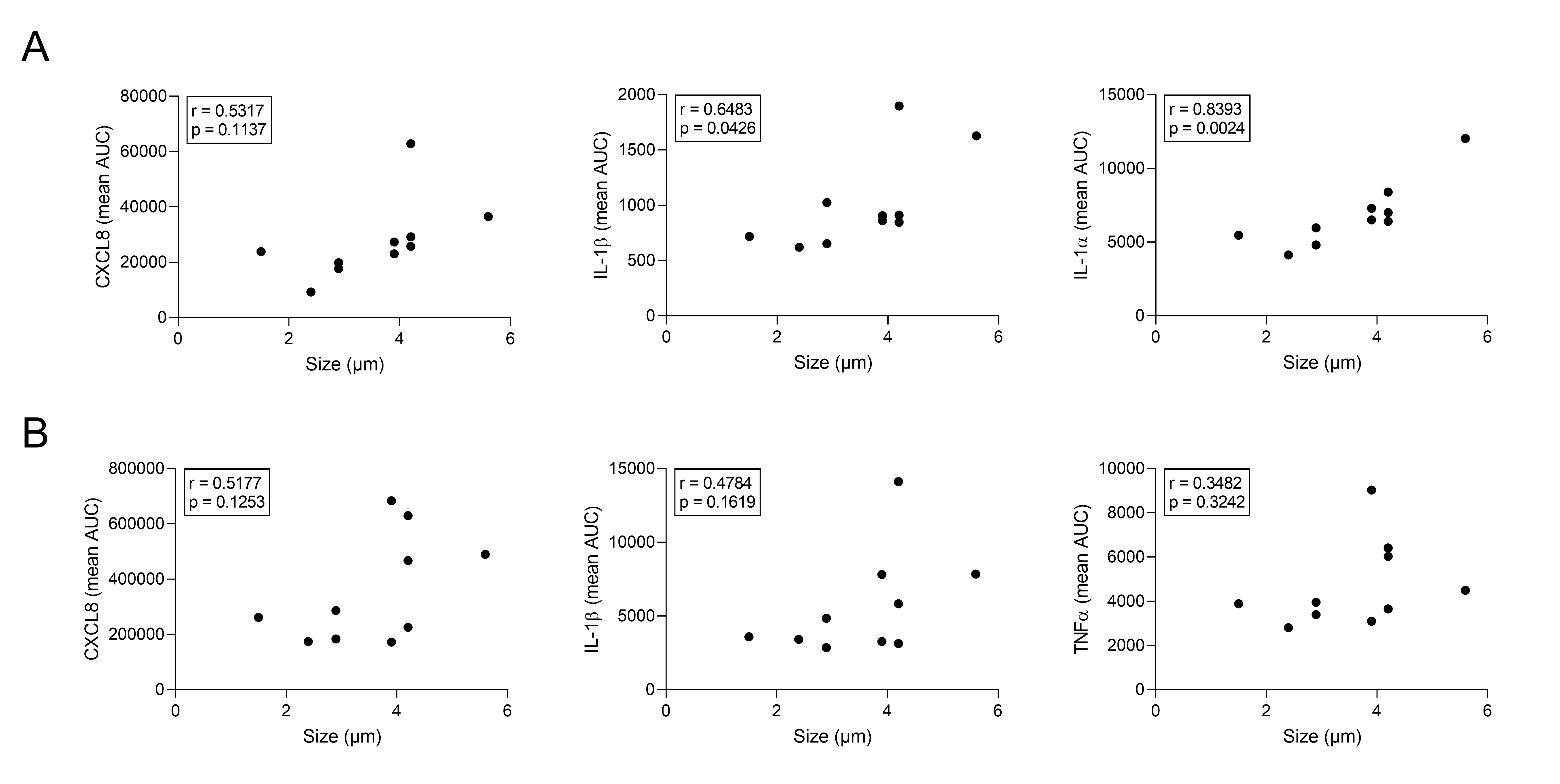

Supplement: Supplementary file 3 — Additional file 3: Fig. S2. The association between particle size and particle-induced cytokine release. The association between particle-induced cytokine release in HBEC3-KT cells A and THP-1 macrophages B was assessed using linear regression. Mean area under the curve (AUC) values for cytokine release were derived from Figs. 4 and 5, while the particle diameter at 50% cumulative volume for each of the mineral samples was derived from Fig. 2. [file 12989_2022_486_MOESM3_ESM.tif]
